# Supplementary material for: Polymerization of misfolded Z alpha-1 antitrypsin protein lowers CX3CR1 expression in human PBMCs
Source: eLife. 2021 May 18;10:e64881. doi: 10.7554/eLife.64881 (PMC8205483; doi:10.7554/eLife.64881)
Supplement: Transparent reporting form [file elife-64881-transrepform.pdf]

eLIFE
1st Floor
24 Hills Road
Cambridge CB2 1JP, UK
P 01223 855340
W elifesciences.org
T @elife

eLife's transparent reporting form

We encourage authors to provide detailed information *within their submission* to facilitate the interpretation and replication of experiments. Authors can upload supporting documentation to indicate the use of appropriate reporting guidelines for health-related research (see EQUATOR Network), life science research (see the BioSharing Information Resource), or the ARRIVE guidelines for reporting work involving animal research. Where applicable, authors should refer to any relevant reporting standards documents in this form.

If you have any questions, please consult our Journal Policies and/or contact us: editorial@elifesciences.org.

Sample-size estimation

- You should state whether an appropriate sample size was computed when the study was being designed
- You should state the statistical method of sample size computation and any required assumptions
- If no explicit power analysis was used, you should describe how you decided what sample (replicate) size (number) to use

Please outline where this information can be found within the submission (e.g., sections or figure legends), or explain why this information doesn't apply to your submission:

- Information about the sample size that is the number of individuals in the study groups or the number of independent experimental repeats is given in the figure legends.
- We didn´t perform an explicit power analysis. AATD is a rare condition. We included all available ZZ AATD individuals with no symptoms of acute exacerbation or infection independent of clinical severity or treatment.
- We collected as many blood samples as available in the close surrounding of Hannover in order to prepare PBMCs within not more than 6 hours after blood donation.
- With ClinCalc.com (https://clincalc.com/stats/samplesize.aspx) we determined a sample size of 42 individuals in total to give a statistical power of 80 %. The probability alpha for a type-I error was set to 0.05. We assumed that CX3CR1 expressions in the test is reduced down to 35 % compared to the control. The standard deviation was set to 75 %.

Replicates

- You should report how often each experiment was performed
- You should include a definition of biological versus technical replication
- The data obtained should be provided and sufficient information should be provided to indicate the number of independent biological and/or technical replicates
- If you encountered any outliers, you should describe how these were handled
- Criteria for exclusion/inclusion of data should be clearly stated
- High-throughput sequence data should be uploaded before submission, with a private link for reviewers provided (these are available from both GEO and ArrayExpress)

Please outline where this information can be found within the submission (e.g., sections or figure legends), or explain why this information doesn't apply to your submission:

- Biological and technical replicates are defined in materials and methods section and figure legends.
- Data points located outside the whiskers are defined as outliers (see dots in figures 1a, b, and 4a). Outliers were included in all calculations.
- We didn´t present high-throughput sequence data.

Statistical reporting

- Statistical analysis methods should be described and justified
- Raw data should be presented in figures whenever informative to do so (typically when N per group is less than 10)
- For each experiment, you should identify the statistical tests used, exact values of N, definitions of center, methods of multiple test correction, and dispersion and precision measures (e.g., mean, median, SD, SEM, confidence intervals; and, for the major substantive results, a measure of effect size (e.g., Pearson's r, Cohen's d)
- Report exact p-values wherever possible alongside the summary statistics and 95% confidence intervals. These should be reported for all key questions and not only when the p-value is less than 0.05.

Please outline where this information can be found within the submission (e.g., sections or figure legends), or explain why this information doesn't apply to your submission:

- The statistical analysis methods are described and justified in the Materials and Methods section.
- Our clinical data comprise too many biological repeats for the presentation of raw data (N=21, 53, or 38). Instead, we show medians (IQR) as boxplots with whiskers and outliers located outside the whiskers.
- We give source data for all figures in the supplement.
- Results of in vitro investigations were presented in boxplots with whiskers or mean (SD) as bar charts depending on failed or passed test for normal distribution of the values.
- Statistical tests used, number of biological repeats, precision measures (mean, SD, median, IQR) are given in the figure legends.
- p-values for all key results are presented in the figures and in the results section.

(For large datasets, or papers with a very large number of statistical tests, you may upload a single table file with tests, Ns, etc., with reference to sections in the manuscript.)

Group allocation

- Indicate how samples were allocated into experimental groups (in the case of clinical studies, please specify allocation to treatment method); if randomization was used, please also state if restricted randomization was applied
- Indicate if masking was used during group allocation, data collection and/or data analysis

Please outline where this information can be found within the submission (e.g., sections or figure legends), or explain why this information doesn't apply to your submission:

- Allocation to AATD group was dependent on the genotype: Only individuals homozygous for Z-AAT variant were included.
- Basis for the recruitment into non-AATD group was a normal plasma AAT levels.

Additional data files ("source data")

- We encourage you to upload relevant additional data files, such as numerical data that are represented as a graph in a figure, or as a summary table
- Where provided, these should be in the most useful format, and they can be uploaded as "Source data" files linked to a main figure or table
- Include model definition files including the full list of parameters used
- Include code used for data analysis (e.g., R, MatLab)
- Avoid stating that data files are "available upon request"

Please indicate the figures or tables for which source data files have been provided:

Source data for all figures are provided.
